# The pregnane xenobiotic receptor, a prominent liver factor, has actions in the midbrain for neurosteroid synthesis and behavioral/neural plasticity of female rats

**DOI:** 10.3389/fnsys.2014.00060

**Published:** 2014-04-21

**Authors:** Cheryl A. Frye, Carolyn J. Koonce, Alicia A. Walf

**Affiliations:** ^1^Department of Psychology, The University at Albany-SUNYAlbany, NY, USA; ^2^Department of Biological Sciences, The University at Albany-SUNYAlbany, NY, USA; ^3^The Center for Neuroscience Research, The University at Albany-SUNYAlbany, NY, USA; ^4^The Center for Life Sciences Research, The University at Albany-SUNYAlbany, NY, USA; ^5^Department of Chemistry and Biochemistry, The University of Alaska–FairbanksFairbanks, AK, USA; ^6^Institute of Arctic Biology, The University of Alaska–FairbanksFairbanks, AK, USA; ^7^IDeA Network of Biomedical Excellence (INBRE), The University of Alaska–FairbanksFairbanks, AK, USA

**Keywords:** midbrain ventral tegmental area, allopregnanolone, progesterone, cognition, reproduction

## Abstract

A novel factor of interest for growth/plasticity in the brain is pregnane xenobiotic receptor (PXR). PXR is a liver factor known for its role in xenobiotic clearance and cholesterol metabolism. It is expressed in the brain, suggesting a potential role for plasticity, particularly involving cholesterol-based steroids and neurosteroids. Mating induces synthesis of neurosteroids in the midbrain Ventral Tegmental Area (VTA) of female rodents, as well as other “plastic” regions of the brain, including the hippocampus, that may be involved in the consolidation of the mating experience. Reducing PXR in the VTA attenuates mating-induced biosynthesis of the neurosteroid, 5α-pregnan-3α-ol-20-one (3α,5α-THP). The 18 kDA translocator protein (TSPO) is one rate-limiting factor for 3α,5α-THP neurosteroidogenesis. The hypothesis tested was that PXR is an upstream factor of TSPO for neurosteroidogenesis of 3α,5α-THP in the VTA for lordosis, independent of peripheral glands. First, proestrous rats were administered a TSPO blocker (PK11195) and/or 3α,5α-THP following infusions of PXR antisense oligonucleotides (AS-ODNs) or vehicle to the VTA. Inhibiting TSPO with PK11195 reduced 3α,5α-THP levels in the midbrain and lordosis, an effect that could be reversed with 3α,5α-THP administration, but not AS-ODN+3α,5α-THP. Second, proestrous, ovariectomized (OVX), or ovariectomized/adrenalectomized (OVX/ADX) rats were infused with a TSPO enhancer (FGIN 1-27) subsequent to AS-ODNs or vehicle to the VTA. PXR AS-ODNs blocked actions of FGIN 1–27 for lordosis and 3α,5α-THP levels among proestrous > OVX > OVX/ADX rats. Thus, PXR may be upstream of TSPO, involved in neurosteroidogenesis of 3α,5α-THP in the brain for plasticity. This novel finding of a liver factor involved in behavioral/neural plasticity substantiates future studies investigating factors known for their prominent actions in the peripheral organs, such as the liver, for modulating brain function and its augmentation.

## Introduction

Steroid hormones are well-recognized for their role for growth processes in the body. For example, physiological roles of steroids hormones for growth are apparent during puberty, with the onset of the patterned secretion of these steroids from the gonads, and morphological differences (secondary sex characteristics), and during pregnancy, with substantial changes in many systems modulated by steroid hormones (e.g., the progestogens—progesterone and its metabolites), as two examples. In these two examples there are obvious physical changes, but the effects, mechanisms, and brain targets of steroids, such as the progestogens, for cognitive and behavioral processes, in relation to the body, are starting to become better understood. Data from large clinical trials conducted of hormone replacement therapies (HRTs) (which typically include synthetic compounds to mimic hormones lost during menopause, estradiol and progesterone), do not entirely support the basic literature on the beneficial role of these hormones for brain functions, like enhancements in learning/memory (described below) and reduction in stroke- and injury-related pathology (Roof et al., 1992, 1993; Chen et al., 1999; He et al., 2004; Shapiro, 2006; Billeci et al., 2007). Moreover, these trials were halted early due to increased cardiovascular and cancer risks and there was a subsequent backlash against clinical use of HRTs that did not take into account individual patient characteristics and risk (Rossouw et al., 2002; Shumaker et al., 2003; Maki and Henderson, 2012). Together, these examples substantiate the importance of understanding the role and mechanisms of steroids in the body and brain.

In addition to the controversy of whether the potential benefits of hormones outweigh their risks, there has been controversy of the role of progestogens for benefiting brain function *per se*. Historically, the basic experimental design to understand whether a particular hormone was necessary and sufficient for a physiological process was to assess co-variation in the hormone of interest and a process and remove the gland that produced the hormone (extirpation) and replace back the hormone (to see if the process is abrogated and then reinstated, respectively). In rodent models of cognitive function, studies assessing natural increases in progestogens over the estrous cycle, or during and after pregnancy, support the mnemonic effects of these high, physiological levels of progestogens in spatial and object recognition tasks (Lambert et al., 2005; Walf et al., 2006; Kinsley and Lambert, 2008; Paris and Frye, 2008; Macbeth and Luine, 2010). Moreover, ovariectomy (surgical removal of the ovaries) of young, adult female rats produces performance deficits in the object recognition and placement tasks, which can be abrogated with replacement back with progesterone or its neuroactive metabolite, 5α-pregnan-3α-ol-20-one (3α,5α-THP, a.k.a. allopregnanolone; Walf et al., 2006; Frye et al., 2007). However, these beneficial effects of progestogens are not uniformly observed in other studies, using different tasks, or dosing and formulation of progestogens, or involving investigations in aged rodents (Murphy et al., 2002; Toung et al., 2004; see review by Acosta et al., 2013). Together, these data suggest that progestogens' effects for cognitive function may be influenced by task, dosing and formulation of progestogen administered, age and likely many other factors.

Given that there are many factors that can influence responses to progestogens for neural processes, an approach that we have taken is to use the well-characterized behavioral model of reproductive behavior of rodents to investigate progestogens' mechanisms. The notion is that we can begin to understand the mechanisms of progestogens in regions that have been investigated for decades (hypothalamus and midbrain) for this basic, hormone-dependent behavior (lordosis, or mating posture of female rodents), substantiating subsequent studies on such mechanisms in corticolimbic structures underlying complex cognitive processes. Lordosis only occurs in appropriate neuroendocrine and environmental context, with reductions in the response supported by effects of ovariectomy, steroid blockers or environmental stressors (Uphouse et al., 2005, 2013; reviewed in Frye, 2011; Frye et al., 2013). From studies using this approach, the effects of progesterone and/or its metabolite, 3α,5α-THP, in the midbrain ventral tegmental area (VTA), through novel neurotransmitter targets (e.g., GABA, dopamine, glutamate, and second messenger cascades) have been supported (reviewed in Frye and Walf, 2008). Interestingly, there is high expression of factors involved in the metabolism and synthesis of 3α,5α-THP in the VTA (from precursor of all steroids, cholesterol; reviewed in Frye, 2011), suggesting the importance of 3α,5α-THP production and action in this region.

Another consideration is that experience can alter hormone levels (and thereby modify the CNS). An example of this is mating-induced neurosteroid synthesis. Mating induces synthesis of neurosteroids in the midbrain VTA of female rodents, as well as other “plastic” regions of the brain, including the hippocampus and prefrontal cortex, that may be involved in the consolidation of the mating experience in rodents. There can be dynamic changes in 3α,5α-THP production in midbrain and corticolimbic structures following mating or other social and environmental challenges (Purdy et al., 1991; Barbaccia et al., 2001; Agís-Balboa et al., 2007; Pinna et al., 2008; Pinna and Rasmusson, 2012). In further support, midbrain 3α,5α-THP levels are higher after female rats are tested in the paced mating task, compared to standard mating task (which does not involve females temporally controlling the frequency of contacts with males), compared to no mating (reviewed in Frye, 2011). Thus, our model system is to use lordosis as a bioassay to further understand mechanisms involved in 3α,5α-THP synthesis and actions for neural/behavioral plasticity.

Studies focused on mechanistic questions about progestogens' actions and production in the VTA, using lordosis as the bioassay, have been extended to understand novel targets, such as the pregnane xenobiotic receptor (PXR), for these responses. PXR has a well-established role as a ubiquitous and promiscuous nuclear receptor in the liver and other excretory organs (kidneys, intestines) for metabolism and xenobiotic clearance (Geick et al., 2001; Dussault and Forman, 2002; Francis et al., 2002; Kliewer et al., 2002). Its role in the CNS was not understood until more recently. Some of the first studies examining PXR's role were those focused on its function in the blood-brain-barrier, which was similar to other excretory organs in the body (Bauer et al., 2004, 2006; Xu et al., 2005; Harmsen et al., 2007; Ma et al., 2008; Zhang et al., 2008; Ott et al., 2009). However, PXR is also expressed in the brain itself, suggesting a potential role for plasticity, particularly involving steroids (which are cholesterol-based hormones) and neurosteroids (which are produced from metabolism of cholesterol in the central nervous system itself). For example, for synthesis of 3α,5α-THP, cholesterol is transported into the mitochondria by way of the 18-kDa translocator protein (TSPO) on the outer mitochondrial membrane. Inside the mitochondria, cholesterol interacts with steroidogenic acute regulatory protein and cytochrome P450-dependent side chain cleavage (P450scc) enzymes (Mellon and Deschepper, 1993; King et al., 2002; Papadopoulos et al., 2006), as well downstream steroidogenic enzymes (5α-reductase, 3α-hydroxysteroid dehydrogenase) to produce pregnenolone, progesterone, dihydroprogesterone, and, ultimately, 3α,5α-THP. A question is the role of PXR in this pathway. Our interest in PXR as a novel target was supported by a microarray study in which the gene for this receptor was expressed in the midbrain following paced mating of female rodents (reviewed in Frye, 2011). We subsequently characterized the expression of this receptor in the midbrain across the estrous cycle of rats (in which we found higher expression associated with higher progestogen levels; Frye et al., 2012, 2013). Moreover, knocking down PXR expression in the VTA reduces mating-induced biosynthesis of 3α,5α-THP in the VTA. Given that TSPO is one rate-limiting factor for 3α,5α-THP neurosteroidogenesis, a question is whether metabolism at TSPO involves, or requires, PXR for production of 3α,5α-THP. The hypothesis was that PXR is a necessary upstream factor of TSPO for neurosteroidogenesis of 3α,5α-THP in the VTA for lordosis, independent of peripheral glands. We assessed the role of PXR, and TSPO manipulations in different hormonal contexts (i.e., over the estrous cycle with extirpation of the ovaries and/or adrenal glands). First, proestrous rats were administered a TSPO blocker (PK11195) and/or 3α,5α-THP following infusions of PXR antisense oligonucleotides (AS-ODNs) or vehicle to the VTA in Experiment 1. We aimed to determine the extent to which the TSPO inhibitor may have effects similar to PXR knockdown and whether this may be reversed with 3α,5α-THP replacement. Second, proestrous (Experiment 2), ovariectomized (OVX; Experiment 3), or ovariectomized/adrenalectomized (OVX/ADX; Experiment 4) rats were infused with a TSPO enhancer (FGIN 1–27) subsequent to AS-ODNs or vehicle to the VTA. We aimed to determine the extent to which the TSPO enhancer may reverse effects of PXR knockdown and whether this may be related to hormonal milieu. Results from these experiments supported the notion that PXR (traditionally considered a liver factor), may be upstream of TSPO, acting as a homeostatic regulator involved in neurosteroidogenesis of 3α,5α-THP in the brain and behavioral plasticity.

## Materials and methods

### Subjects and housing

Adult, Long–Evans female rats (*n* = 236), approximately 55 days of age, were bred in the Life Sciences Laboratory Animal Care Facility at The University at Albany–SUNY (original stock: Taconic Farms, Germantown, NY, USA) and/or shipped from Taconic. Rats were group-housed in polycarbonate cages with woodchip bedding (45 × 24 × 21 cm) in a temperature- (21 ± 1°C) and humidity- (50 ± 5%) controlled room in the Laboratory Animal Care Facility. Rats were maintained on a 12:12 h reversed light cycle (lights off at 0800 hours) with continuous access to Purina Rat Chow and tap water in their home cages.

### General procedure

**Figure 1:**
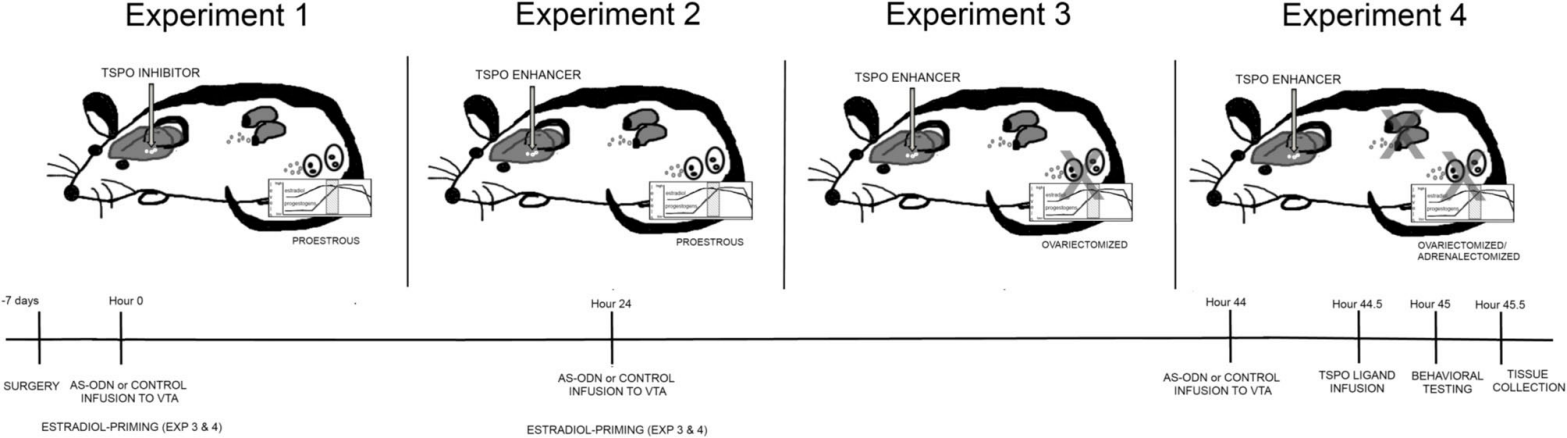
**Depicts different hormonal conditions and TSPO ligand infusions in rats (top) and timeline of experimental protocol (bottom).** In Experiment 1, all rats were in the proestrous phase of the estrous cycle and administered via infusions to the VTA, placebo or PXR AS-ODNs (at hour 0, 24, and 44), followed by an inhibitor of TSPO (PK11195) or saline vehicle and/or 3α,5α-THP (at hour 44.5). In Experiment 2, all rats were in the proestrous phase of the estrous cycle and administered via infusions to the VTA, placebo or PXR AS-ODNs (at hour 0, 24, and 44), followed by an enhancer of TSPO (FGIN) or saline vehicle (at hour 44.5). In Experiment 3, all rats were ovariectomized (OVX) and administered subcutaneous injections of E_2_ at hour 0 and 24 and then administered via infusions to the VTA, placebo or PXR AS-ODNs (at hour 0, 24, and 44), followed by an enhancer of TSPO (FGIN) or saline vehicle (at hour 44.5). In Experiment 4, all rats were OVX and adrenalectomized (OVX/ADX), and administered subcutaneous injections of E_2_ at hour 0 and 24 and then administered via infusions to the VTA, placebo or PXR AS-ODNs (at hour 0, 24, and 44), followed by an enhancer of TSPO (FGIN) or saline vehicle (at hour 44.5). All rats were tested at hour 45, and euthanized for tissue collection immediately after behavioral testing was complete. In all experiments, knockdown of PXR expression in the VTA was validated and levels of 3α,5α-THP were measured in the midbrain.

Four experiments were run, using a between- and within-subjects design. All experimental rats had stereotaxic surgery to implant cannulae aimed at the VTA, 7 days before drug manipulations were initiated. Experimental rats were randomly-assigned to one treatment condition (between-subjects) within a single experiment, and then were tested in a single battery of behavioral tasks (within-subjects). The general procedure utilized is depicted in Figure 1. All experimental techniques in rats were approved by the Institutional Animal Care and Use Committee at the University at Albany, where the experiments were conducted.

### Surgical manipulations

All rats in Experiments 1–4 were stereotaxically implanted with bilateral guide cannulae aimed at the VTA (from bregma: AP = −5.3, ML = ±0.4, DV = −7.0; Paxinos and Watson, 1986) under xylazine (12 mg/kg) and ketamine (80 mg/kg) anesthesia per prior methods (Frye et al., 2013). Immediately following stereotaxic surgery, rats were OVX (*n* = 49; Experiment 3) and/or OVX/ADX (*n* = 48; Experiment 4). For OVX/ADX, dorsal incisions were made between the ribs and hip, so that the ovaries and the adrenals could be isolated. The ovaries were ligated and removed, and the adrenals were isolated and removed. As ADX rats are sodium deficient, all rats received a bottle of 0.9% saline and a bottle of tap water in their home cages to maintain water/salt balance. Following surgery and prior to testing, animals were monitored for loss of weight, righting response, flank stimulation response, and/or muscle tone. Only rats that passed neurological evaluations and gained weight following surgery until behavioral testing commenced were continued in the experiment. All rats that underwent ADX were validated to have complete ADX surgery via *post-hoc* assessment of corticosterone in plasma. Rats were administered ibuprofen (once per oral, and daily in the recovery period in drinking water; 30 mg/kg) as post-surgical analgesia.

### Estrous cycle

Vaginal epithelium of each rat was collected and examined daily under a light microscope (between 0800–1000). Rats were cycled through two normal estrous cycles (4–5 days cycle) prior to testing. Rats were tested on proestrus (epithelium characterized by nucleated cells, 4–5 days after the previous occurrence) for Experiments 1 (*n* = 68) and 2 (*n* = 71).

### Infusions

For Experiments 1–4, rats received either sterile saline (0.9% w/v) or PXR AS-ODN infusions. AS-ODNs by (5′ CTTGCGGAAGGGGCACCTCA 3′; made in a concentration of 100 ng/μl; Frye et al., 2012, 2013, 2014, in press) were synthesized and desalted Invitrogen Life Technologies (Carlsbad, CA). This PXR AS-ODN strategy decreases expression of PXR in the midbrain, as measured by quantitative polymerase chain reaction (qPCR) (which was utilized here to validate) and western blotting (Frye, 2011; Frye et al., 2012, 2013). In Experiment 1, PK11195 (Tocris Biosciences, R&D Systems, Minneapolis, MN) was utilized as a TSPO antagonist (compounded by adding crystalline drug to sterile saline at a concentration of 50 ng per μl; Bitran et al., 2000; Frye et al., 2006, 2009). 3α,5α-THP (purchased from Dr. Robert Purdy, Scripps Institute, CA) was prepared to a concentration of 100 ng per μl in β-cyclodextran (with 5% concentration of β-cyclodextran in sterile water; Frye and Rhodes, 2006; Frye et al., 2008a, 2014). In Experiments 2–4, a neurosteroidogenesis enhancer, FGIN 1–27, an agonist of TSPO was utilized (Petralia and Frye, 2005; Frye et al., 2009, used in Experiments 2–4). Crystalline FGIN was compounded in a concentration of 50 ng per μl (Tocris) in sterile saline. Rats received bilateral infusions of each compound or placebo vehicle. This infusion protocol has been utilized without any indication of damaging effects as assessed by behavioral changes, or estradiol levels (given damage can induce aromatase activity, and thereby local estradiol levels; Azcoitia et al., 2003); this was determined by comparing rats that were sham surgerized, had stereotaxic implantation of cannulae to the VTA, but were not administered infusions, and those that were administered infusions of saline vehicle in the same volumes as drugs are administered (Frye et al., 2013).

### Estradiol priming

For Experiment 3 and 4, OVX and OVX/ADX rats were primed with subcutaneous administration of estradiol(10 μg in 0.2 cc vegetable oil) at 0 and 24 h before behavioral testing.

### Behavioral measures

Our primary interest was in the role of PXR and TSPO for reproductive responding (as measured in the paced mating task); however, control measures of exploration, anxiety, and social interaction were also collected. Behavioral data were simultaneously collected by using the Any-maze behavioral assessment computer program (Stoelting Inc., Wood Lawn, IL; for open field, elevated plus maze, and social interaction) or a digital video camera (for paced mating) and trained experimenters.

#### Control measures- exploration, anxiety, non-sexual social interaction

Immediately before assessment in the paced mating task, rats were tested sequentially in the open field for 5 min, the elevated plus maze for 5 min, and the social interaction tasks, as per established methods (see Frye et al., 2013). Across experiments, there were no differences between comparisons groups in rats that were tested for exploratory behavior in the open field, anxiety behavior in the elevated plus maze, or social interaction with a female conspecific.

#### Paced mating

Paced mating was conducted per previous methods (Erskine, 1985; Frye et al., 2013) in an apparatus (37.5 × 75 × 30 cm) that was divided down the center by a Plexiglas partition. An experimental female has access to both sides of the pacing chamber, while a stimulus male was confined to one side for a 15-min test period. Standard measures of mating behavior (lordosis quotients, proceptivity quotients, and aggression quotients) are being reported herein. Lordosis quotients are the percentage of total number of lordosis responses per total number of sexual contacts by the male. Aggression/rejection (aggression quotients) are defined as behaviors such as boxing and kicking the male during contacts.

### Tissue collection and preparation

Immediately after testing, rats were euthanized by rapid decapitation and whole brain and trunk blood was collected, frozen, and stored in a freezer. For brain dissections, punches from the midbrain, around the VTA, were taken from coronal frozen slices (approximately 60 microns thick), made anterior and posterior to the VTA, and used for analyses of PXR expression (described below). At this time of collecting these slices, whether cannulae/infusion tracks were aimed at the VTA was determined (as per Frye et al., 2013, 2014). There were a total of *n* = 17 rats that had placement outside of the VTA; their data was excluded from analyses of the data from rats with placement to the VTA. Not all experimental groups were represented in rats that had missed sites, precluding systematic analyses of the data from these rats to those with placement to the VTA. However, in comparing the data available, the pattern that emerged was that rats with placement outside of the VTA had responses similar to control groups.

### Validation of PXR knockdown following PXR AS-ODNs

Standard qPCR methods were utilized on VTA punches to determine whether PXR AS-ODN infusions reduced PXR expression (described in Frye et al., 2013, 2014). Data were analyzed by comparing PXR values to actin control, and PXR expression is described as fold-change of the rats infused with PXR AS-ODNs to those infused with control (saline; Livak and Schmittgen, 2001; Schmittgen and Livak, 2008; Frye et al., 2013, 2014).

### Steroid hormone measurement

Plasma levels of corticosterone, estradiol, and progesterone were measured to validate adequacy of ADX, estrous cycle stage and/or OVX and hormone-priming, respectively. Concentrations of corticosterone, estradiol, progesterone and 3α,5α-THP were assessed using standard steroid extraction and radioimmunoassay (primarily for corticosterone and 3α,5α-THP) or commercially-available enzyme-linked immunosorbent assays (estradiol, progesterone) techniques used by our laboratory (Frye and Bayon, 1999; Frye et al., 2008a, 2013, 2014, in press). Concentrations were determined for each sample based upon concurrent standard curves run in duplicate for each of the assays. Concentrations reflect the approximate concentrations of the volume of plasma, or protein concentrations determined in midbrain homogenate samples measured by a NanoDrop spectrometer.

### Statistical analyses

Analyses of variances (ANOVAs) were used to examine effects of PXR AS-ODN condition (control, PXR AS-ODN), PK11195 condition (control, PK11195) and 3α,5α,-THP condition (β-cyclodextrin, 3α,5α,-THP for Experiment 1) and for Experiment 2–4, PXR AS-ODN condition (control, PXR AS-ODN) and FGIN condition (control, FGIN), and/or testing condition (non tested, tested) on behavioral and endocrine measures. The assumptions of homogeneity of variance were supported, suggesting that parametric ANOVA tests be utilized as the most appropriate statistic across experiments. When the α level for statistical significance was reached (*p* = 0.05) for main effects and interactions, Fisher's Least Significant Differences *post-hoc* tests were used to determine group differences.

## Results

### Experiment 1: effects of TSPO blocker, PK11195, in proestrous rats

#### Validation of PXR AS-ODNs for PXR expression in the midbrain

Rats infused with PXR AS-ODN had 1.5 fold lower PXR expression in the midbrain VTA compared to rats infused with control condition [*F*_(1, 29)_ = 11.9, *P* < 0.001].

#### 3α,5α-THP levels in the midbrain (Figure 2A)

There was significant interaction between PXR AS-ODN condition and 3α,5α-THP condition [*F*_(1, 60)_ = 10.7, *P* < 0.002] for 3α,5α-THP levels in the midbrain VTA, such that control rats infused with 3α,5α-THP had higher levels of 3α,5α-THP levels in the midbrain compared to PXR AS-ODN infusions. There were significant main effects of PXR AS-ODN [*F*_(1, 60)_ = 29.7, *P* < 0.0001], 3α,5α-THP [*F*_(1, 60)_ = 13.1, *P* = 0.0007], and PK11195 [*F*_(1, 60)_ = 7.3, *P* = 0.01] conditions for 3α,5α-THP levels in the midbrain. Infusions of PXR AS-ODNs reduced, 3α,5α-THP increased, and PK11195 reduced 3α,5α-THP levels in midbrain compared to control infusions for each manipulation.

**Figure 2:**
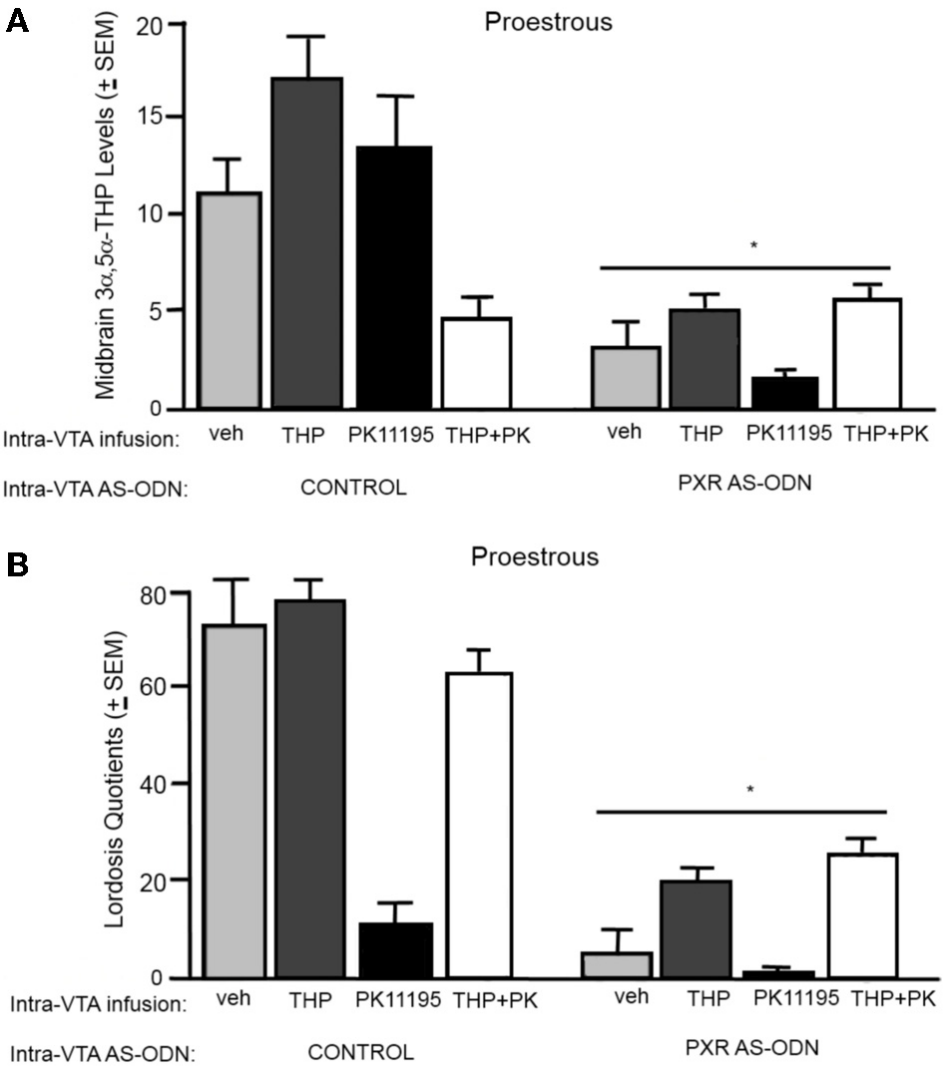
**(A)** Depicts the mean (+s.e.m.) 3α,5α-THP levels in the midbrain of proestrous rats infused first with control and vehicle (veh, n = 5), 3α,5α-THP (*n* = 7), PK11195 (*n* = 12), PK11195+3α,5α-THP (*n* = 11), or pregnane xenobiotic receptor (PXR) antisense deoxynucleotides (AS-ODNs) and veh (*n* = 10), PXR AS-ODN +3α,5α-THP (*n* = 8), PXR AS-ODN+ PK11195 (*n* = 7), PXR AS-ODN + PK11195+ 3α,5α-THP (*n* = 8) in Experiment 1. **(B)** Depicts the mean (+s.e.m.) lordosis quotient of proestrous rats in these conditions. Rats infused with PXR AS-ODN had a mean 1.5 fold lower PXR expression in the midbrain compared to control infusions in Experiment 1 (data not shown). ^*^above a line indicates a significant effect of PXR AS-ODNs compared to control infusion (*P* < 0.05). ^*^above a bar indicates a significant effect of PK11195 and PK1195+3α,5α-THP compared to vehicle infusions (*P* < 0.05).

#### Lordosis quotients (Figure 2B)

There was an interaction between 3α,5α-THP condition and PK11195 condition [*F*_(1, 60)_ = 11.4, *P* = 0.001] for lordosis quotients, such that PK11195 reduced lordosis quotients, but not when co-administered with 3α,5α-THP. There was significant interaction between PXR AS-ODN condition and 3α,5α-THP condition [*F*_(1, 60)_ = 4.7, *P* = 0.03] for lordosis quotients, such that control rats infused with 3α,5α-THP had increased lordosis quotients, but not when co-administered PXR AS-ODNs. There was a significant main effect of PXR AS-ODN condition [*F*_(1, 60)_ = 12.5, *P* = 0.0008] to reduce lordosis quotients compared to those infused with control.

#### Aggression quotients (Table 1)

There was significant main effect of PXR AS-ODN condition [*F*_(1, 60)_ = 19.9, *P* = 0.0003] and PK11195 condition [*F*_(1, 60)_ = 3.9, *P* = 0.05] for aggression quotients. Rats infused with PXR AS-ODN or PK11195 had increased aggression quotients compared to respective control infusions.

**Table 1:** **Depicts mean (± s.e.m.) aggression quotients (AQ) of rats during the paced mating task In Experiments 1–4**.

**EXPERIMENT 1**										
Hormone condition	Proestrous									
AS-ODN condition	Control					PXR AS-ODN				
Infusion condition	Veh	3α,5α-THP	PK11195		PK11195 + 3α,5α-THP	Veh	3α,5α-THP	PK11195		PK11195 + 3α,5α-THP
AQ	33.9 ± 10.6	14.4^ˆ^ ± 8.8	49.2 ± 9.1		12.5 ± 5.1	51.8^*^ ± 18.2	37.9^*^^ˆ^ ± 7.5	58.3^*^ ± 8.3		46.4^*^ ± 9.7
**EXPERIMENT 2**										
Hormone condition	Proestrous									
AS-ODN condition	Control					PXR AS-ODN				
FGIN condition	Veh			FGIN		Veh			FGIN	
AQ	26.5 ± 9.0			30.9 ± 7.6		34.2 ± 12.6			46.5 ± 9.2	
**EXPERIMENT 3**										
Hormone condition	OVX, E_2_-primed									
AS-ODN condition	Control					PXR AS-ODN				
FGIN condition	Veh			FGIN		Veh			FGIN	
AQ	40.6 ± 11.6			74.2 ± 10.4		75.2 ± 12.1			66.2 ± 7.9	
**EXPERIMENT 4**										
Hormone condition	OVX/ADX, E_2_-primed									
AS-ODN condition	Control					PXR AS-ODN				
FGIN condition	Veh			FGIN		Veh			FGIN	
AQ	39.6 ± 12.9			23.0 ± 10.1		61.1^*^ ± 10.3			66.6^*^ ± 16.0	

* Indicates a significant difference of pregnane xenobiotic receptor (PXR) PXR antisense oliodeoxynucleotides (AS-ODN) from control (P > 0.05).

ˆ Indicates significant interaction between AS-ODN, PK11195, and 3 α, 5 α-THP (P > 0.05).

### Experiment 2: effects of TSPO enhancer, FGIN 1–27, in proestrous rats

#### Validation of PXR AS-ODNs for PXR expression in the midbrain

Rats infused with PXR AS-ODN had 1.8 fold lower PXR expression in the midbrain VTA compared to rats administered control infusions [*F*_(1, 37)_ = 36.4, *P* < 0.0001].

#### 3α,5α-THP levels in the midbrain (Figure 3A)

There was a significant main effect of PXR AS-ODN condition [*F*_(1, 52)_ = 14.4, *P* = 0.0004] for 3α,5α-THP levels in the midbrain. Rats infused with PXR AS-ODN had significantly lower 3α,5α-THP in the midbrain compared to those infused with control; effects of testing and FGIN infusions did not reach statistical significance.

**Figure 3:**
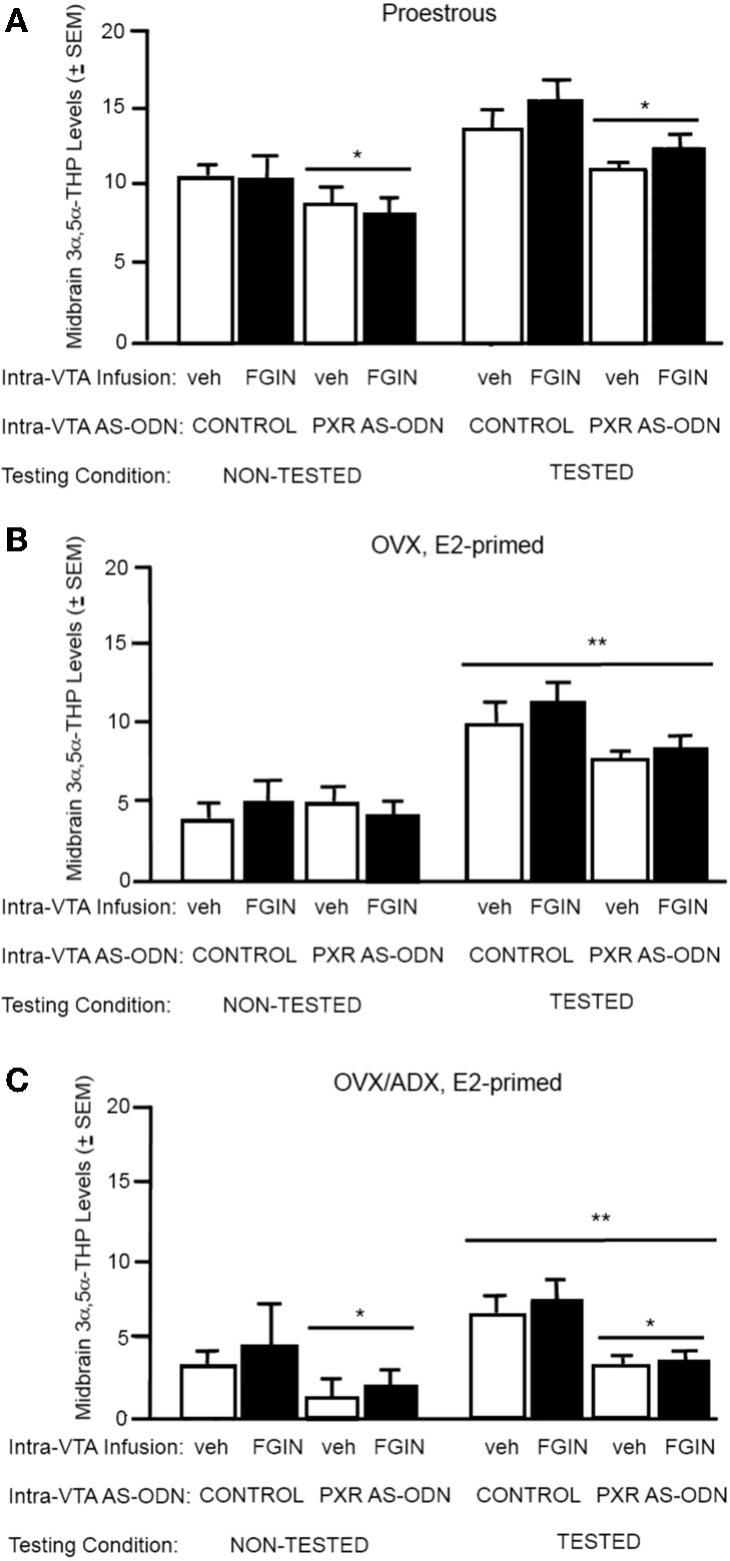
**(A)** Depicts the mean (±s.e.m.) 3α,5α-THP levels in the midbrain of proestrous non tested rats infused with control and vehicle (veh, *n* = 5) or control+FGIN (*n* = 4), or pregnane xenobiotic receptor (PXR) antisense deoxynucleotides (AS-ODNs) and veh (*n* = 6), or PXR AS-ODN+FGIN (*n* = 6) or behaviorally-tested rats infused with control+veh (*n* = 15), control+FGIN (*n* = 15), PXR AS-ODN+veh (*n* = 8), or PXR AS-ODN+FGIN (*n* = 13) in Experiment 2. Rats infused with PXR AS-ODN had a mean 1.8 fold lower PXR expression in the midbrain compared to control infusions in Experiment 2 (data not shown). **(B)** Depicts the mean (±s.e.m.) 3α,5α-THP levels in the midbrain of ovariectomized (OVX) estradiol (E2)-primed non tested rats infused with control+veh (*n* = 5), control+FGIN (*n* = 6), PXR AS-ODN+veh (*n* = 5), PXR AS-ODN+FGIN (*n* = 5), or behaviorally-tested rats infused with control+veh (*n* = 8), control+FGIN (*n* = 7), PXR AS-ODN+veh (*n* = 7), or PXR AS-ODN+FGIN (*n* = 6) in Experiment 3. Rats infused with PXR AS-ODN had a mean 1.5 fold lower PXR expression in the midbrain compared to control infusions in Experiment 3 (data not shown). **(C)** Depicts the mean (±s.e.m.) 3α,5α-THP levels in the midbrain of OVX/adrenalectomized (ADX), E2-primed rats that were non tested and infused with control+veh (*n* = 6), control+FGIN (*n* = 5), PXR AS-ODN+veh (*n* = 5), PXR AS-ODN+FGIN (*n* = 5), or behaviorally-tested and infused with control+veh (*n* = 9), control+FGIN (*n* = 5), PXR AS-ODN+veh (*n* = 8), or PXR AS-ODN+FGIN (*n* = 5) in Experiment 4. Rats infused with PXR AS-ODN had a mean 1.4 fold lower PXR expression in the midbrain compared to control infusions in Experiment 4 (data not shown). ^*^above a line indicates a significant effect of PXR AS-ODNs compared to control. ^**^above a line indicates significant effect of testing compared to non-tested rats (*P* < 0.05).

#### Lordosis quotients (Figure 4A)

There was a significant interaction between PXR AS-ODN condition and FGIN condition [*F*_(1, 47)_ = 3.8, *P* < 0.05] for lordosis quotients, such that PXR AS-ODNs reduced lordosis, but not when co-administered with FGIN. There was a significant main effect of PXR AS-ODN condition [*F*_(1, 47)_ = 3.8, *P* = 0.05] for lordosis quotients. Rats infused with PXR AS-ODN had significantly lower lordosis quotients compared to those infused with control condition.

**Figure 4:**
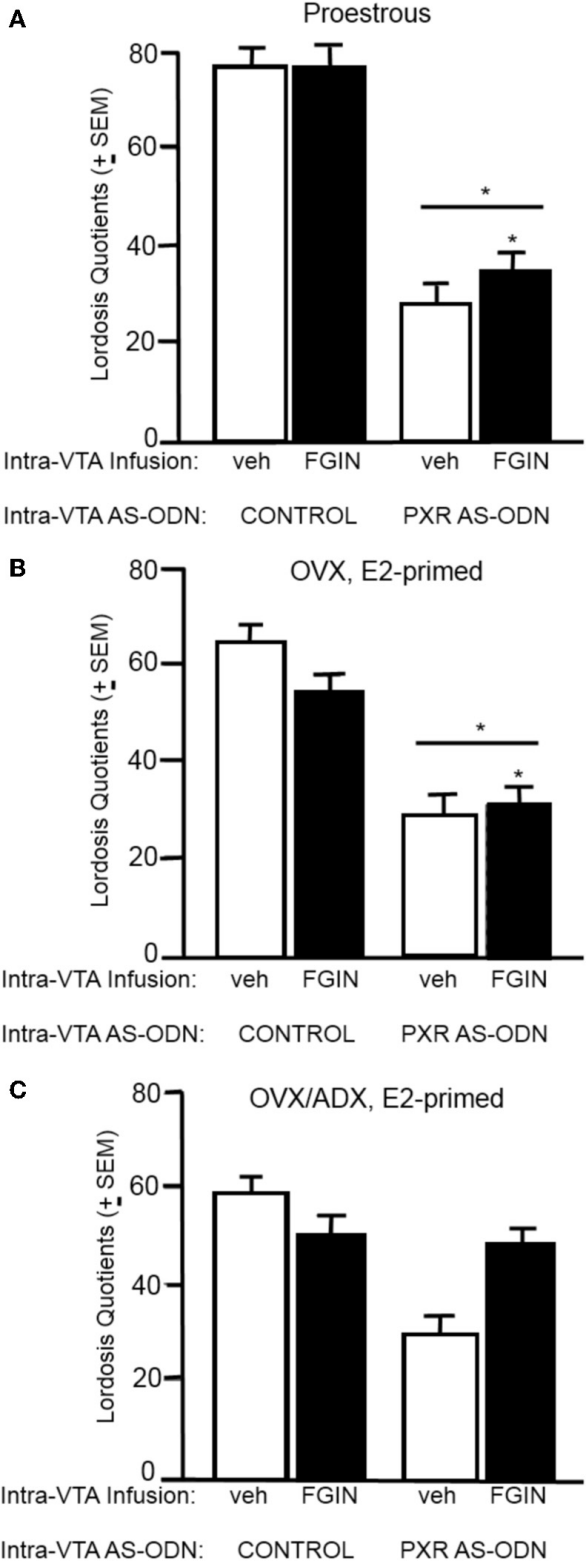
**(A)** Depicts the mean (±s.e.m.) lordosis quotients of proestrous rats infused with control and vehicle (veh, *n* = 15), control+FGIN (*n* = 15), pregnane xenobiotic receptor (PXR) antisense deoxynucleotides (AS-ODNs)+vehicle (*n* = 8), or PXR AS-ODN+FGIN (*n* = 13) in Experiment 2. **(B)** Depicts the mean (±s.e.m.) lordosis quotients of ovariectomized (OVX) estradiol (E2)-primed rats infused with control+veh (*n* = 8), control+FGIN (*n* = 7), PXR AS-ODN+veh (*n* = 7), or PXR AS-ODN+FGIN (*n* = 6) in Experiment 3. **(C)** Depicts the mean (±s.e.m.) lordosis quotients of OVX/adrenalectomized (ADX), E2-primed rats infused with control+veh (*n* = 9), control+FGIN (*n* = 5), PXR AS-ODN+veh (*n* = 8), or PXR AS-ODN+FGIN (*n* = 5) in Experiment 4. ^*^above a line indicates a significant effect of PXR AS-ODNs compared to control. (*P* < 0.05).

#### Aggression quotients (Table 1)

There were no statistically significant effects of conditions for aggression quotients in this experiment.

### Experiment 3: effects of TSPO enhancer, FGIN 1–27, in OVX rats

#### Validation of PXR AS-ODNs for PXR expression in the midbrain

Rats infused with PXR AS-ODN had 1.5 fold lower PXR expression in the midbrain VTA compared to control infusions [*F*_(1, 41)_ = 14.2, *P* = 0.0005].

#### 3α,5α-THP levels in the midbrain (Figure 3B)

There was a main effect for testing condition [*F*_(1, 41)_ = 16.9, *P* = 0.002] for 3α,5α-THP levels in the midbrain, such that tested rats had higher levels of 3α,5α-THP in midbrain compared to non-tested rats; effects of PXR AS-ODN and FGIN infusions did not reach statistical significance.

#### Lordosis quotients (Figure 4B)

There was a significant main effect of PXR AS-ODN condition [*F*_(1, 24)_ = 18.9, *P* = 0.0002] and FGIN condition [*F*_(1, 24)_ = 9.3, *P* = 0.005] for lordosis quotients. Rats infused with PXR AS-ODN had significantly lower, and those infused with FGIN had significantly higher, lordosis quotients, compared to those infused with respective control conditions.

#### Aggression quotients (Table 1)

There were no significant main effects for AS-ODN condition or FGIN condition for aggression quotients.

### Experiment 4: effects of TSPO enhancer, FGIN 1–27, in OVX/ADX rats

#### Validation of PXR AS-ODNs for PXR expression in the midbrain

Rats infused with PXR AS-ODN had 1.4 fold lower PXR expression in the midbrain VTA compared to control infusions [*F*_(1, 24)_ = 6.4, *P* = 0.01].

#### 3α,5α-THP levels in the midbrain (Figure 3C)

There was main effect of testing condition [*F*_(1, 38)_ = 10.3, *P* = 0.002] and PXR AS-ODN condition [*F*_(1, 38)_ = 4.4, *P* = 0.04] for 3α,5α-THP levels in the midbrain. Tested rats had higher levels of 3α,5α-THP in midbrain compared to non-tested rats, and rats infused with PXR AS-ODNs had lower levels compared to control infusions.

#### Lordosis quotients (Figure 4C)

There was main effect of PXR AS-ODN condition [*F*_(1, 25)_ = 5.8, *P* = 0.02] for lordosis quotients, such that infusions of PXR AS-ODN decreased lordosis quotients compared to control infusions; effects of FGIN infusions did not reach statistical significance.

#### Aggression quotients (Table 1)

There was main effect of PXR AS-ODN condition [*F*_(1, 25)_ = 4.9, *P* = 0.03] for aggression quotients, such that infusions of PXR AS-ODN decreased aggression quotients compared to control infusions; effects of FGIN infusions did not reach statistical significance.

## Discussion

The data support, in part, the hypothesis tested that PXR is an upstream factor of TSPO for neurosteroidogenesis of 3α,5α-THP in the VTA for lordosis, independent of peripheral glands. First, proestrous rats administered a TSPO blocker (PK11195), subsequent to AS-ODNs to the VTA, had lower 3α,5α-THP levels in the midbrain and lordosis; the effects on lordosis could be reversed with replacement with 3α,5α-THP administration alone, but not when co-administered with PXR AS-ODN. Second, proestrous, OVX, or OVX/ADX rats infused with a TSPO enhancer (FGIN 1–27), subsequent to AS-ODNs to the VTA, had lower 3α,5α-THP levels in the midbrain and lordosis particularly among proestrous rats, compared to those that were OVX or OVX/ADX. Comparisons with removal of the main peripheral sources of steroids, ovaries and adrenals, were completed to address the role of PXR and TSPO for steroid synthesis in the brain. These data with a greater response in proestrous compared to rats with glands surgically removed suggest that there is not complete independence of these peripheral sources for PXR's actions in the midbrain VTA, which are likely upstream of TSPO. Thus, PXR may be acting as a homeostatic regulator, upstream of TSPO in the pathway for production of 3α,5α-THP in the midbrain VTA, and behavioral responses of female rats.

The present data confirm the importance of PXR in the midbrain for 3α,5α-THP and lordosis. Inhibiting PXR with the same AS-ODN approach used here reduces 3α,5α-THP in the midbrain VTA concomitant with reductions in lordosis and enhancements in aggressive responding toward males during paced mating of proestrous rats, or those that were OVX and estradiol-primed (Frye et al., 2013, 2014). Additionally, activating PXR with ligands infused to the midbrain VTA enhances reproductive responding of female rats (Frye, 2011). Moreover, the present data confirm the role of biosynthesis of 3α,5α-THP from cholesterol in the midbrain VTA for lordosis. Inhibitors of TSPO, as well as downstream factors involved in biosynthesis, StAR or P450scc, infused to the midbrain VTA of female rats attenuates progestogen-facilitated lordosis (Petralia et al., 2005; Frye et al., 2008a; Frye, 2009; Frye and Paris, 2011). An opposite effect is observed with TSPO agonist, such as FGIN, when infused to the midbrain VTA as was observed here (Frye et al., 2009; Frye and Paris, 2011).

It should be noted that FGIN infusions in this study did not produce the same pattern of reduction for 3α,5α-THP levels in the midbrain as was observed in lordosis quotients. A consideration is that this experiment was conducted in proestrous rats, with peripheral sources of steroids, as well as typical steroid feedback mechanisms in place; a more robust response may have been observed with this FGIN dosing in OVX and/or OVX/ADX rats. Another consideration is that comparisons with non-tested rats in this experiment may have provided an indication of mating-induced 3α,5α-THP production from metabolism of progesterone from the ovaries, which could account for little difference in 3α,5α-THP levels in the midbrain after mating, albeit low lordosis responding with this manipulation. A third consideration is that measurement of dihydroprogesterone, or other steroids, were not assessed in the midbrain. Indeed, in the group that was co-administered PK11195 and 3α,5α-THP, the enhancements in lordosis were more robust than post-mating levels of 3α,5α-THP on the midbrain that were measured; whether this could have been due to back-conversion of administered 3α,5α-THP or 3α,5α-THP from other sources is not known. Together, these data extends previous findings to suggest that PXR, which is known to act as a transcription factor for P450 enzymes involved in steroid metabolism, such as CYP11A1 (Ma et al., 2008; Zhang et al., 2008), may be another important neuroregulatory factor in the biosynthesis of 3α,5α-THP in the midbrain, upstream of TSPO, and resulting actions on lordosis in the adult. Moreover, the data that there were differences between proestrous, OVX, and OVX/ADX rats suggest that biosynthesis in the brain, as well as metabolism from peripheral sources of progesterone, are involved in PXR's modulation of 3α,5α-THP. Alternatively, the removal of the glands disrupts multiple negative feedback loops of steroids, and thereby disrupts typical responses to steroids for behavior. We have observed that effects of PXR AS-ODN infusions to reduce lordosis and 3α,5α-THP levels in the midbrain of OVX rats only when they were estradiol-primed (Frye et al., 2014). Thus, hormonal milieu provides a significant context for interactions between neuroendocrine and behavioral responses.

The pattern of results here confirms prior studies showing a greater influence of PXR knockdown in the midbrain for behavior in the paced mating task, compared to the other behavioral tasks assessed (even when the order of testing in these tasks is altered as in Frye et al., in press). There were very few animals that received infusions outside the targeted area, and all experimental groups were not represented. The data from these subjects suggest that manipulations to other midbrain sites (e.g., substantia nigra, or central gray) did not produce the same pattern of effects as did infusions to the VTA, but rather produced responses similar to the control groups. Manipulations of 3α,5α-THP from exposure to mating, or infusions of 3α,5α-THP directly to this the midbrain (but not substantia nigra or central gray), increase levels of 3α,5α-THP in corticolimbic structures and reduce anxiety-like responding; however, manipulations of PXR typically produce less robust effects on these other measures, as was observed here. A question for future studies is the expression patterns and actions of PXR beyond the midbrain for reproduction-relevant behaviors, such as cognition, exploration, anxiety, and interactions with conspecifics. Mating-induced neurosteroidogenesis occurs beyond the midbrain to the hippocampus, cortex, and striatum (Frye and Rhodes, 2006; Frye et al., 2009). Indeed, we have observed that manipulating PXR in the midbrain of proestrous rats reduces 3α,5α-THP levels in the midbrain and the hippocampus, as well as a growth factor (brain-derived neurotrophic factor; BDNF) in the hippocampus (Frye et al., 2013, in press). It is plausible that PXR is having effects beyond those involving progestogen-facilitated lordosis through actions in the midbrain.

Beyond facilitating successful mating, progestogens are considered to have organizing effects on the nervous system during gestation/perinatally to influence later adult behaviors that ultimately are adaptive (reducing stress/anxiety, enhancing cognition, and conferring protection to neural insults/aging; Frye, 2009; Brinton, 2013; Brunton et al., 2014; Bali and Jaggi, 2014). We have focused on using lordosis as a bioassay to understand progestogens' mechanisms and effects in the central nervous system; albeit, other models support a role of progestogens for motivated, and cognitive processes. One example is mating-induced conditioned placed preference, where females will change their initial preference to spend time in a context associated with mating (González-Flores et al., 2004; Camacho et al., 2009; Arzate et al., 2011). Beneficial effects of progesterone or 3α,5α-THP administration to ovariectomized rats or mice has been described for spatial and object recognition memory tasks (Sandstrom and Williams, 2001; Tanabe et al., 2004; Walf et al., 2006; Frye et al., 2007, 2013). Furthermore, the capacity for *de novo* steroid synthesis in the prefrontal cortex and hippocampus (Cheng and Karavolas, 1975; Li et al., 1997; Furukawa et al., 1998; Frye, 2001a,b) suggests that PXR may also be a factor to investigate systematically in these regions for behaviors that contribute to successful mating. For example, in recent studies assessing the requirement of PXR for mating-induced 3α,5α-THP synthesis in the midbrain and the hippocampus, knocking down PXR attenuated effects of paced mating experience to increase 3α,5α-THP in these regions, as well as increase BDNF in the hippocampus, of proestrous rats (Frye et al., in press). Given the role of progestogens throughout the lifespan, the importance of PXR-mediated 3α,5α-THP production and other measures of neural plasticity (e.g., BDNF) and behavioral plasticity (e.g., mating) are of continued interest.

In addition to further understanding potential downstream factors, such as TSPO and BDNF, the contributory role of other “liver” factors in the same nuclear receptor super family as PXR, such as liver X receptor (LXR), for neural and behavioral plasticity needs to be explored. Although the present experiment focused on the role of PXR, it would be of interest to consider the respective roles of these factors, which were first described in the liver, but both have received a greater focus on their effects in the nervous system more recently (Mellon et al., 2008). One focus of the effects of these receptors is their role in cholesterol clearance, which is related to diet and cardiovascular function, as well as important for neurodegenerative disorders, such as Alzheimer's disease, and neurodevelopmental disorders, such as Niemann-Pick disease (Whitney et al., 2002; Repa et al., 2007; Ma et al., 2008; Tang et al., 2008). As one example, mouse models of Niemann-Pick disease show deficits in cholesterol metabolism and 3α,5α-THP production in the brain, and such deficits could be reversed with administration of PXR ligands, one of them being 3α,5α-THP (Frye and Rhodes, 2006; Frye, 2009; Brinton, 2013).

Early developmental effects of LXR in the brain have also been described. For example, protective effects similar to those described for PXR have been noted with LXR modulation in a Niemann-Pick disease mouse model (Repa et al., 2007) as well as promoting neurosteroidogenesis and protective effects in animal models of diabetic neuropathy and multiple sclerosis (Cermenati et al., 2010, 2012; Mitro et al., 2012). As well, the role of LXR in typical neurodevelopmental processes, such as neurogenesis, in the VTA of mice, using an *in vitro* model, has been recently described (Theofilopoulos et al., 2013). Additionally, knockout of the beta form of LXR that is highly expressed in the central nervous system increases anxiety-like responding of female mice and alters GABA in the cortex (Tan et al., 2012). In the present study, the role of PXR in the VTA for 3α,5α-THP production from cholesterol and subsequent behavioral effects was assessed. A consideration for future studies is elucidating how PXR and LXR may act synergistically for efficient cholesterol metabolism and clearance in neurons and glia, respectively, supporting neural and behavioral plasticity. Another important question is the role of PXR for neurosteroidogenesis in males; production of androstane neurosteroids, such as 3α-androstanediol, occurs after mating of male rodents and has robust behavioral actions (Edinger and Frye, 2007; Frye et al., 2008b). Although proestrous female rats have higher expression of PXR in the midbrain than do males (Frye et al., 2013), the expression of PXR in males and females in other brain regions with high capacity for neurosteroidogenesis is not completely known. Together, these results involving liver factors, such as PXR, substantiate further studies of an interaction between peripheral and brain factors for behavior.

In summary, inhibiting TSPO with PK11195 reduced 3α,5α-THP levels in the midbrain and lordosis, an effect that could be reversed with 3α,5α-THP administration, but not AS-ODN+3α,5α-THP. PXR AS-ODNs blocked actions of FGIN 1–27 for lordosis and 3α,5α-THP levels among proestrous > OVX > OVX/ADX rats. Together, these data suggest the liver factor, PXR, may be upstream of TSPO, acting as a homeostatic regulator involved in neurosteroidogenesis of 3α,5α-THP in the brain for behavior. Understanding these basic mechanisms of how steroids are involved, and what factors are necessary for their production, for behavioral/neural plasticity supports future work on how a prominent peripheral factor, such as PXR, may have actions in the brain and, ultimately, find use in brain augmentation approaches.

## Author contributions

All authors on this paper substantially contributed to the work described herein. Carolyn J. Koonce was involved in acquisition, analysis, and interpretation of data, and drafting of figures for paper and Results and Methods section. Alicia A.Walf was involved in acquisition, analysis, and interpretation of data, and drafting and revising of all sections of the paper. Cheryl A. Frye was involved in the conception and study design, acquisition, analysis, and interpretation of data, reviewing drafts of the work, and giving final approval of the paper to be submitted.

### Conflict of interest statement

The authors declare that the research was conducted in the absence of any commercial or financial relationships that could be construed as a potential conflict of interest.
